# Characterization and clinical evaluation of a novel IMRT quality assurance system

**DOI:** 10.1120/jacmp.v10i2.2928

**Published:** 2009-05-07

**Authors:** Ramaswamy Sadagopan, Jose A. Bencomo, Rafael L. Martin, Gorgen Nilsson, Thomas Matzen, Peter A. Balter

**Affiliations:** ^1^ Department of Radiation Physics The University of Texas M. D. Anderson Cancer Center Houston Texas U.S.A.; ^2^ Department of Physics Universidad Central de Venezuela Caracas Venezuela; ^3^ ScandiDos Uppsala Sweden

**Keywords:** IMRT, quality assurance, 3D measurement, 4D measurement, diodes

## Abstract

Intensity‐modulated radiation therapy (IMRT) is a complex procedure that involves the delivery of complex intensity patterns from various gantry angles. Due to the complexity of the treatment plans, the standard care is to perform measurement‐based, patient‐specific quality assurance (QA). IMRT QA is traditionally done with film for relative dose in a plane and with an ion chamber for absolute dose. This is a laborious and time‐consuming process. In this work, we characterized, commissioned, and evaluated the QA capabilities of a novel commercial IMRT device, Delta^4^, (ScandiDos, Uppsala, Sweden). This device consists of diode matrices in two orthogonal planes inserted in a cylindrical acrylic phantom that is 22 cm in diameter. Although the system has detectors in only two planes, it provides a novel interpolation algorithm that is capable of estimating doses at points where no detectors are present. Each diode is sampled per beam pulse so that the dose distribution can be evaluated on segment‐by‐segment, beam‐by‐beam, or as a composite plan from a single set of measurements. The end user can calibrate the system to perform absolute dosimetry, eliminating the need for additional ion chamber measurements. The patient's IMRT plan is imported into the device over the hospital LAN and the results of the measurements can be displayed as gamma profiles, distance‐to‐agreement maps, dose difference maps, or the measured dose distribution can be superimposed on the patient's anatomy to display an as‐delivered plan. We evaluated the system's reproducibility, stability, pulse‐rate dependence, dose‐rate dependence, angular dependence, linearity of dose response, and energy response using carefully planned measurements. We also validated the system's interpolation algorithm by measuring a complex dose distribution from an IMRT treatment. Several simple and complex isodose distributions planned using a treatment planning system were delivered to the QA device; the planned and measured dose distributions were then compared and analyzed. In addition, the dose distributions measured by conventional IMRT QA, which uses an ion chamber and film, were compared. We found that the Delta4 device is accurate and reproducible and that its interpolation algorithm is valid. In addition, the supplied software and network interface allow a streamlined IMRT QA process.

PACS number: 87.56Fc

## I. INTRODUCTION

In intensity‐modulated radiation therapy (IMRT), many small beamlets combine to produce a complex dose distribution. There are many sources of uncertainty in the IMRT process chain including: treatment planning system (TPS) commissioning measurements; approximations and limitations in calculation algorithms;^(^ ^1^ ^–^ ^3^ ^)^ the delivery process, especially multileaf collimator (MLC) calibrations;^(^ ^3^ ^–^ ^5^ ^)^ performance of the accelerator in the low monitor unit (MU) setting;^(6)^ ^,^ ^(7)^ patient information; and verification of measurements.^(^ ^8^ ^–^ ^12^ ^)^ Therefore, diligent patient‐specific quality assurance (QA) is mandatory with IMRT.^(^ ^13^ ^,^ ^14^ ^)^ However, IMRT QA is laborious, time consuming, and may not be the best use of limited resources in a busy clinical setting.

Currently, IMRT QA is typically performed using ionization (ion) chambers for absolute‐dose verification, and film for relative‐dose verification of the composite plan in a single plane. The ion chamber method is regarded as the gold standard in conventional radiation measurements; however, the volume‐average effects produced by this methodology renders it less ideal for IMRT QA.^(12)^ Films, such as EDR2 (Eastman‐Kodak, Rochester, NY), are also limited QA tools in that they: 1) provide fine spatial resolution for the dose gradients encountered in IMRT fields but lack the ability to perform real‐time measurements; 2) are not reproducible due to film processor variations;^(15)^ 3) show intermittency^(15)^ and time delay between exposure and processing effects;^(16)^ 4) show directional and energy dependence;^(17)^ and 5) even with frequent calibrations, provide only relative dose distribution. When measurements are made using ion chambers or film, further processing of the raw data is required to complete the QA analysis. Furthermore, patient‐specific QA using one of these techniques is usually performed in a two‐dimensional (2D) spatial plane, yet neither technique is easily extended to dynamic treatments such as gating or tumor tracking for motion management^(18)^, and neither offers assistance in identifying the source of error without additional beam‐by‐beam measurements.

A major concern in using film to perform IMRT QA is that it is a 2D dosimeter and this may not give sufficient information to ensure a quality treatment is delivered. If the film measurements are done in a single transverse plane, the performance of the delivery outside this plane may not be captured. Hansen et al. and Das et al.^(6)^ ^,^ ^(7)^ reported the performances of various linear accelerators with several photon energies in low MU settings (<5 MU). They demonstrated that the output, fatness, and symmetry need not be the same as those measured in standard MU settings (e.g., 100 MU). In addition, some MLC leaves may never be evaluated if they are outside the plane of measurement. Other methods of IMRT verification include MU calculations,^(^ ^19^ ^–^ ^21^ ^)^ Monte Carlo calculations,^(^ ^2^ ^,^ ^22^ ^,^ ^23^ ^)^ use of an electronic portal imaging device (EPID) for fluence verification and for exit‐beam dosimetry,^(^ ^24^ ^,^ ^25^ ^)^ diode array^(26)^, and ion chamber array.^(27)^ ^,^ ^(28)^ However, MU and Monte Carlo calculations do not address delivery errors.^(29)^ As well, EPIDs, diode arrays, and ion chamber arrays do not address leaf sag unless performed at various gantry angles; clinical relevance of any discrepancies found would be difficult to assess due to lack of geometrical correlation.^(24)^ EPID‐based QA offers excellent spatial resolution but requires correction factors for energy dependence.^(30)^ Létourneau et al.^(26)^ evaluated a 2D diode array (MapCHECK from Sun Nuclear, Melbourne, FL) for absolute and relative quality assurance measurements for IMRT. They showed that MLC calibration error of 1 mm can be detected even though the diodes were spaced at 7.1 mm. Other commercially available systems for IMRT verification include the PTW seven29 Matrix (Laco, Inc., Chesterland, OH), and I'mRT MatriXX (IBA Dosimetry GmbH, Schwarzenbruck, Germany).^(28)^ These systems are primarily designed to measure dose or fluence perpendicular to the incident beam direction and, while a composite dose distribution could be generated by summing multiple beams, this approach lacks the verification of gantry, collimator, and couch angles and does not correlate the measured composite dose distribution to the patient anatomy. Hence, the clinical impact of the IMRT QA result, such as uncertainty in the maximum cord dose region, was not clearly determined.

**Figure 1:**
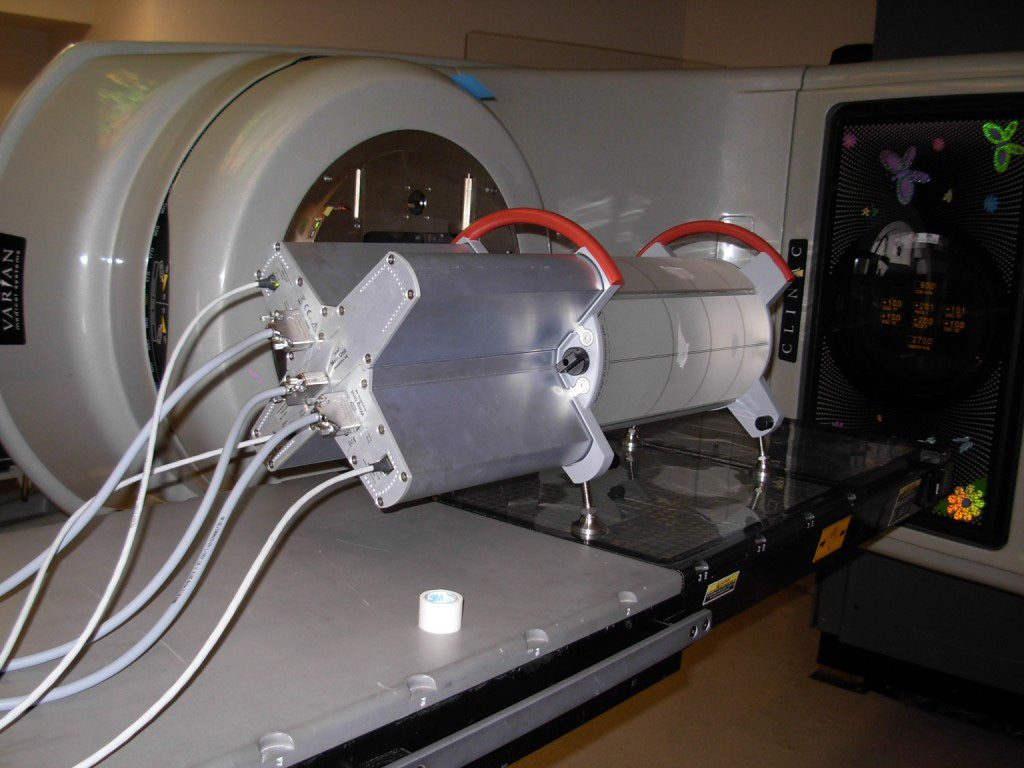
Delta^4^ phantom in the measurement position. 1069 detectors are built in two orthogonal planes with a resolution of 0.5 cm in the center and 1 cm at the periphery. Each diode is a cylinder of 0.5 mm diameter and height of 0.05 mm.

A novel IMRT QA device, Delta^4^ (ScandiDos AB, Uppsala, Sweden), overcomes many of the shortcomings of other methods. Delta^4^ employs 1,069 diodes in two orthogonal planes enclosed in a cylindrical phantom (Fig. 1). The device synchronizes the pulses of the linear accelerator and stores readings on a pulse‐by‐pulse basis, facilitating time‐based analysis. It also imports the planned beam geometry, the dose distribution of the plan delivered to the phantom geometry, and, if desired, the patient structures for evaluation purposes. The planned dose distribution is used to estimate a 3D dose distribution based on ray lines passing through one of the two detector planes. The results of the measurements can be presented as either distance‐to‐agreement maps, dose difference maps, gamma values, or an overlay of “as‐delivered” dose onto the contours of the planning computed tomography (CT). The “time‐resolved” feature of this device allows the analysis to be displayed as a composite or as a beam‐by‐beam – or even segment‐by‐segment – analysis from a single set of measurements.

In this study, we characterized, commissioned, and evaluated the Delta^4^ device. The detectors were assessed using measurements obtained under carefully designed experiments. Our objective was to evaluate the suitability of the system in the clinic and to understand its capabilities and limitations. The evaluation of the system was performed by IMRT QA measurements of several clinical treatment plans.

## II. MATERIALS AND METHODS

### A. Delta4 IMRT QA System

Delta^4^ consists of 1069 p‐type diodes arranged in a matrix along two orthogonal planes. The sensitivity of the p‐type semiconductor is less subject to the effects of radiation than that of the n‐type semiconductors.^(31)^ Also, p‐type detectors have been shown to have little saturation of signals, especially at high levels of dose per pulse, compared with n‐type detectors.^(31)^ Each p‐type diode has a cylindrical sensitive volume with a 0.78 mm^2^ area and a thickness of 0.05mm. The detectors are spaced at 0.5 cm intervals in the central 6 cm×6 cm area and at 1 cm intervals outside of this area, and they cover an area of 20 cm×20 cm. The detector planes are placed in an acrylic cylindrical phantom 22 cm in diameter and 40 cm in length (Fig. 1). The detector planes subtend angles of 50° clockwise and 40° counter‐clockwise with respect to the vertical axis. This is done to avoid beams being aligned with the detector planes The software will instruct the operator to irradiate the phantom with the 40° plane to the left or right, based on the treatment plan being evaluated. Multichannel electrometers are located at the ends of the detector planes in an integrated module. A coaxial cable is run into the treatment room to provide beam synchronization pulses from the accelerator to the electrometers, and a CAT‐5 cable is run out of the room to transfer data to the control computer. The measured data are synchronized with the accelerator pulses and stored on a pulse‐by‐pulse basis, allowing segment‐by‐segment analysis and 4D treatment QA. Although the system has detectors along two orthogonal planes only, the device provides a novel technique for calculating the dose in 3D, which is discussed in the next section.

The cylindrical phantom has double‐line crosshairs inscribed on three sides and leveling screws on four corners, to aid in setup. The double lines are 1.5 mm apart, and the phantom position is adjusted until the room lasers fall between these lines. The system comes with a transfer cart that holds the body of the phantom, leaving the feet protruding. This enables the staff to bring the treatment table up to apply the weight of the phantom on the feet and permits the transfer cart to be removed without any manual lifting of the phantom. This feature is important because, together, the phantom and detector system weigh 27 Kg.

#### A.1 3D Dose Interpolation

**Figure 2:**
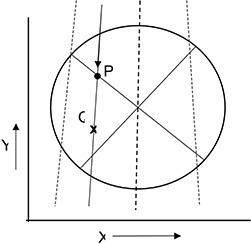
Illustration of the Delta^4^ interpolation algorithm – axial view. Point P represents a detector and Point Q represents a point for which dose needed to be interpolated. If the ray line passes through two detectors, one detector is arbitrarily chosen for the renormalization point.

The 3D dose estimation is accomplished on a beam‐by‐beam basis. The dose contribution from each beam in 3D space is rescaled along a set of ray lines from the source to each detector in the Delta^4^ device (Fig. 2). The dose along that ray line is assumed to scale linearly with the ratio of measured to calculated dose at the detector (Eq. 1). This is done for all beams, and the results are summed to obtain the measured 3D dose distribution. For beams that pass through both detector planes, only one of the detector planes is used for this rescaling. The rescaling can be understood by examining Fig. 2 and Eq. 1. As shown, $D_{M}$(P) and $D_{C}$(P) are the measured dose and calculated dose (from the TPS); then the dose, $D_{\text{SM}}$, that would be measured at point Q is given by the following equation.

(1) $$D_{SM}(Q)=D_{M}(P)*D_{C}(Q)/D_{C}(P)$$

where $D_{\text{SM}}$ denotes “semi‐measured” dose. This approach works because, for a photon beam, the difference in dose between points P and Q is governed by the simple physical principles of inverse square law and exponential attenuation.

We validated this technique for determining 3D dose distributions by comparing a “semi‐measured” 3D dose distribution with physically measured 3D data for a clinical IMRT plan. The method of validation is described later in this paper.

#### A.2 Data Acquisition/Synchronization

All current‐generation medical linear accelerators employ pulsed beams. The Varian 2100 EX linear accelerator (Varian Inc., Palo Alto, Ca) uses a pulse width of 3 μsec with a pulse spacing of 3 msec on the 6‐MV beam. To maximize the signal‐to‐noise ratio, this system takes advantage of the time between the dose pulses to read and re‐zero the measurement diodes. Because the system is not accumulating between pulses, dark current effects are minimized. The total dose is then the sum of the dose/pulse measurements for that beam.

#### A.3 Data Management and Review

All acquired data are stored in a central database. The dose information at any level–segment, beam, or composite of beams – is always available for analysis. As with conventional IMRT QA procedures, a patient's IMRT treatment plan is recalculated substituting the cylindrical phantom for the patient's anatomy. The beam data and the calculated dose distributions are transferred to the Delta^4^ device via DICOM RT and/or the Radiation Therapy Oncology Group (RTOG) format. Because the DICOM dose export has not yet become standard, the RTOG format is supported as an interim solution. Analysis of patients planned on TPS systems that cannot export DICOM dose on a beam‐by‐beam basis requires the export of both the DICOM RT and the RTOG formats, because neither provides a complete data set. Because the system corrects for the small angular dependence of the detectors, no measurements can be performed without a corresponding treatment plan.

There are several display and analysis tools available within the software. The analysis tools include: (a) comparison of measured and calculated profiles in 2D, (b) a beam's eye view (BEV) display of the measured intensity pattern along with deviations of measured leaf positions from the calculated values, (c) histograms of dose deviations, (d) dose‐to‐distance agreement, and (e) gamma analysis histograms. The measured dose can also be displayed as isodose contours overlaid with patient structures. The display feature includes the ability to exclude detectors that receive a below‐threshold dose (out‐of‐field) from the final analysis. The analysis is interactive, permitting the clinician to change dose or distance to agreement (DTA) criteria in real time with instant updates of the analysis.

### B. Calibrating the Delta4 System

Calibration of the detector arrays involves two steps: absolute calibration of the central detector, and relative calibration of all the detectors. The absolute calibration needs to be done for each beam energy on each type of linear accelerator. The relative calibration only needs to be done once. Both of these calibrations are done with detectors removed from the cylindrical phantom and placed in a rectangular acrylic slab phantom.

Absolute dose is determined for a $10\times 10\;{\text{cm}}^{2}$ reference condition using an ion chamber in the same phantom that will be used for normalizing the detector arrays. The ionization chamber is replaced by the detector arrays, and the absolute sensitivity of the central detector is determined.

The relative sensitivity is determined in the same phantom but with a field size large enough to encompass the detector arrays. The cross calibration process involves irradiating and then translating the arrays. This is repeated seven times with translations in both directions perpendicular to the beam in order to crosscalibrate the detectors in a stable beam.

### C. Characterization of the Delta4 System

Characterization was performed by measuring the following parameters: short‐ and long‐term reproducibility, linearity with dose, dose rate (pulse repetition rate), and dose per pulse. In addition, directional dependence, response to scatter, and sensitivity to leakage radiation were also evaluated. Many of the characterization measurements were performed with the detector planes inside the cylindrical phantom using a 6‐MV beam from a Varian 2100 EX linear accelerator at a constant repetition rate of 400 MU/min, except where indicated.

#### C.1 Short and Long‐term Reproducibility

The short‐term reproducibility of the system was evaluated in a single session by measuring the dose from a single 6‐MV beam ten times, using an open‐field size of 20 cm×20 cm and examining the diodes within the central 80% of the field. The long‐term reproducibility of the system was evaluated in two ways. The first method involved measuring a box‐shaped dose distribution from four 20 cm×20 cm fields, five times over a 3‐month period. Each time, the accelerator output was verified using a Farmer‐type chamber in a solid phantom. The 4‐field box distribution was chosen to reduce uncertainty introduced in the measurement due to set‐up errors. The second method involved estimating the decrease in sensitivity of the diode signal due to radiation damage from the calibrations of the diodes against an ionization chamber performed at two different times.

#### C.2 Linearity, Dose Rate and Dose‐per‐pulse Response

The linearity of the system was measured for a single 20 cm×20 cm field size using 50 MU to 1000 MU. The diode dose rate dependence was studied in two ways: 1) by measuring the dose per MU by changing the pulse repetition frequency on the console, and 2) by measuring the diode response as a function of dose per pulse. The diode response for a given dose rate was measured and compared for the entire range of the available pulse repetition frequencies from 100 MU/min to 600 MU/min. The dose per pulse was varied by changing the source‐to‐detector distance, and the diode readings were measured at various dose‐per‐pulse values. The rectangular acrylic phantom with the detector at a 1.5 cm depth was used for this experiment. The source‐to‐detector distance ranged from 77 cm to 159 cm. The dose was measured using a Farmer‐type chamber at each detector distance. The chamber's collection efficiency was also measured to correct the chamber readings at each distance. Care was taken to scale down the field size as the source‐to‐detector distance was increased in order to keep the phantom scatter constant. It has been shown that the type of diode used in the device slightly over responds to phantom scatter at very large field sizes and depths.^(32)^

#### C.3 Angular Response

The angular or directional dependence of the diodes was measured as a function of beam angles. The detector arrays exhibit some angular dependence, but this is corrected automatically based on the known orientations of the beam with respect to the detector planes. In order to understand the magnitude of these corrections, we irradiated the phantom from a number of gantry angles. For each of these irradiations, the Delta^4^ system used the same reference plan that contained a single zero degree beam. Due to the cylindrical geometry of the phantom, the same dose was delivered to the isocenter for all beams; therefore, the differences in the reported dose should be the uncorrected angular dependence of the system.

#### C.4 Response to Scatter and Leakage Radiations

The response of the detector to scatter and leakage radiation was evaluated by measuring and comparing the ratio of near out‐of‐field dose (scatter dose) to central‐axis dose. MLC leakage factors were also measured using a CC04 ionization chamber (IBA, Schwarzenbruck, Germany) for both 6‐MV and 18‐MV beams. The CC04 chamber was chosen because its energy response is similar to tissue as its walls and central electrode are made of Shonka C554 tissue‐equivalent plastic. The scatter dose was measured in the acrylic phantom at the depths of the respective $d_{\max}$ of the beams with sufficient backscatter. The field sizes, defined by the MLC at isocenter, ranged from 2 cm×2 cm to 10 cm×10 cm. For each field, the photon jaws were set 1 cm larger than the MLC field in order to minimize the dose contribution from leakage radiation. The ratio of the readings at 1 cm and 2 cm away from the edge of the field to that on the central axis were determined using both the diodes and the ionization chamber. The leakage factors through the MLC were determined by taking the ratio of the measured dose on the central axis for an open field to the one blocked by the MLC. Care was taken to ensure that the leaves were closed behind the jaws and the sensitive volume of the chamber was positioned just below a leaf. The MLC leakage factors were determined for both 6‐MV and 18‐MV beams using a 10 cm×10 cm field size, as defined by the jaws at isocenter.

#### C.5 3D Dose Interpolation

The interpolation method of the device was verified for an IMRT treatment plan having a complex dose distribution by comparing measured values with interpolated values along the same planes. The cylindrical geometry of the phantom enabled us to rotate the entire treatment plan with respect to the detector planes while keeping the shape of the dose distribution intact. First, the dose distribution of an IMRT treatment plan was measured by the device (initial plan). Then, the plan was re‐delivered but with all beams rotated by 10°, then 40°, and then 50°, which was equivalent to rotating the detector planes in the opposite direction. The rotational offsets brought the planes of detectors into regions where no detectors were initially present. The interpolated dose values from the initial plan were extracted. Under normal operation, only measured values can be exported, but a special software patch was supplied by the device manufacturer to facilitate this analysis. Custom software was written in MATLAB (The MathWorks Inc., Natick, MA) that transformed the measured and interpolated matrices to be in the same orientation so that profiles could be compared. The interpolated values from the Delta^4^ system were in a rectilinear grid, where the measured points maintain a radial symmetry. Therefore, the relative spacing of the interpolated nodes with respect to the measurement positions changes as a function of angle.

### D. Evaluation

The device was evaluated for thirteen clinical IMRT treatment plans covering several typical treatment sites: central nervous system, head and neck, lung, esophagus, gastrointestinal, gynecologic, prostate, and rectum. At the start of each measuring session, the ambient temperature was recorded, and the output of the linear accelerator was verified using a Farmer‐type ionization chamber. IMRT plans containing non‐coplanar 6‐MV and 18‐MV beams were specifically included in the evaluation of the device. All beams planned were delivered at the clinical‐use dose (rep) rate of 400 MU/min. All plans were developed with the Pinnacle v7.6c or v8.0 system (Philips, Bothell, WA), and no changes were made in the photon algorithm between these versions. The Pinnacle v7.6c did not allow us to export dose distribution on a beam‐by‐beam basis; therefore, some of the features were not available, such as the planned and measured dose deviation on any axial plane and the display of measured and calculated dose volume histograms. However, these options were tested using the Pinnacle v8.0.

The evaluation included the analysis of the percentage of detectors that passed the ±5% dose criteria and the gamma calculated for 5% dose and 5 mm (5%/5 mm) distance criteria and for 3%/3 mm criteria (Table 1). The dose and gamma criteria were analyzed and presented in two ways: 1) full analysis, when all detector readings were included, and 2) threshold analysis, when detector readings with doses above 20% of the isocenter dose were included.

**Table 1:** Summary the IMRT QA results performed using Delta^4^ system. Values are percent of detectors passing the given criteria.

	*Delta^4^*							*Film*		*Ion*
*S. No*.	*Site*	*Detectors* >20−200% *included*			*Detectors 0–200% included*			*Gamma*, ±5%		*Chamber, Ratio of*
		*Dose*	*% Gamma*		*Dose*	*% Gamma*		*% pixel*	*% pixel*	*measd/calculated*
		±5%	*5%, 5mm*	*3%, 3mm*	±5%	*5%, 5mm*	*3%, 3mm*	≤1.00	>1 <2	
1	Lung	93.5	99.8	94.7	96.0	99.8	95.2	98.6	1.4	0.995
2	Lung	74	95.3	83.4	89.0	97.7	92.9	96.9	3.1	0.992
2	Repeat	77.2	96.4	86.8	90.3	97.8	93.7	na	na	Na
3	Lung	86.9	98.6	81.5	89.2	98.9	84.7	99.6	0.4	1.019
4	Esophagus	94.4	99.9	96.2	96.0	99.8	94.4	99.0	1.0	1.001
5	Esophagus	90.8	99.8	88.1	92.2	99.7	88.7	94.0	6.0	0.989
6	GE Junction	97.2	99.9	98.7	97.5	99.8	98.8	98.8	1.2	1.000
7	Prostate,SV	82.6	98.6	80.6	92.9	99.2	92.0	na	na	na
7	Prostate,SV	58.2	93.6	73.0	85.3	97.0	87.5	99.5	0.5	0.994
8	Prostate,SV	58.9	85.5	62.9	79.9	92.9	81.8	99.5	0.5	0.990
9	Gyn	99.2	99.8	97.6	99.3	99.8	97.8	97.7	2.3	1.004
10	Rectum	95.2	99.3	89.1	95.4	99.0	84.0	93.4	6.6	1.026
11	Head&Neck	61.9	97.5	87.4	60.3	84.8	62.4	93.1	6.9	0.983
12	Brain	90.7	100.0	91.4	94.9	99.7	95.5	100.0	0.1	1.000
13	Brain	53.9	77.8	57.1	72.8	86.8	74.7	98.7	1.3	1.005

## III. RESULTS

### 1. Short and Long‐term Reproducibility

The short‐term reproducibility of the detector response was found to be 0.1% (1 standard deviation, σ), with a range of 0% to 1%, for the 904 diodes evaluated over 10 irradiations. The long‐term reproducibility was found to be 0.5% (1 σ) when measured five times over a 3‐month period. We estimated the change in the detector sensitivity due to radiation damage by comparing the readings per absorbed dose before and after delivering about 800 Gy. The decrease in sensitivity observed was to be about 0.9%kGy for a 6‐MV beam.

### 2. Linearity, Dose Rate and Dose‐per‐Pulse Response

**Figure 3:**
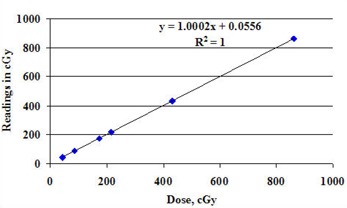
Linearity of the central detector. The uncertainty of these measurements is 1SD=0.2% and smaller than the symbols used for the display.

**Figure 4:**
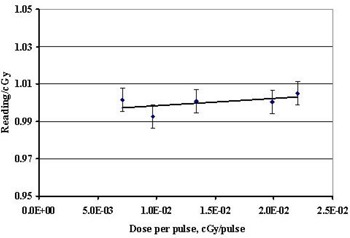
Diode response as function of dose per pulse. The uncertainty displayed represents the uncertainty in the diodes and the uncertainty in the ion chamber measurements added in quadrature (1 σ).

Dose response linearity was measured over a range of 43 cGy to 863 cGy. The response was found to be linear within 0.25%, as shown in Fig. 3. It should be noted that no saturation dose was observed or expected because the charge generated by the diodes was read and reset between each beam pulse. In addition, no dose (rep) rate dependence was observed in the range of 100 MU/min to 600 MU/min. The effects of dose per pulse were also measured by changing the source‐to‐detector distance (Fig. 4). A 3‐fold change in the dose (from 0.35 mGy/pulse to 1.05 mGy/pulse) resulted in less than a 0.25% change in diode response. For the dose/pulse determinations, the uncertainty of the determination was 0.6% (1 s), which represents the combined uncertainty of the diode and ion chamber measurements.

### 3. Angular Response

**Figure 5:**
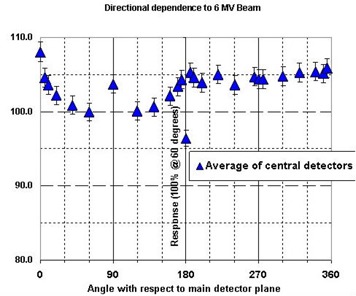
Uncorrected angular response of the detectors as a function of incident beam angle with respect to the main detector plane (detectors along the major axis of the cylinder in the central 80% of field were analyzed). In practice, an angular correction is applied to all measured data. Diode readings were arbitrarily normalized at the 60° angle, and the displayed uncertainty represents ±1 σ. The 0° and 180° readings represent angles that are avoided by the design of the hardware/software system; all other data falls within a ±2.5% range.

The angular dependence of the central diode in the Delta^4^ system was measured (Fig. 5). The uncorrected angular dependence varied by ±2.5%, excluding the gracing angles at 0° and 180° to the large detector plane containing the central diode. The effect of the orthogonal detector plane can also be seen at 90° and 270°. It should be noted that though the angular dependence is symmetrical about the normal of the detector plane, it is not symmetrical about the detector plane itself; this is due to radiation interaction with the printer circuit board from one side.

### 4. Response to Scatter and Leakage Radiations

The response of the detector to scatter and leakage radiation for 6‐MV and 18‐MV beams was also measured (Table 2). Due to the small signals, all data are presented as percentages of the central‐axis dose. The out‐of‐field to central‐axis ratios measured by the diode agreed with those measured by the CC04 ionization chamber within 1% of the central‐axis dose. The leakage factors measured with the two systems were found to be within 0.4% of each other. The uncertainty of both the scatter dose and leakage factor measurements was 0.6% (1σ). Therefore, the energy response characteristics were found to be suitable for IMRT QA measurement.

**Table 2:** Comparison of scatter and leakage measurements obtained with the Delta^4^ and with a CC04 ion chamber. Each value represents a ratio to the unblocked CAX measurement.

*MLC FS [cm^2^]*	*JAW FS [cm^2^]*	*DIST. Field Edge [cm]*	*Delta^4^ 6 MV*	*CC04 6 MV*	*Delta^4^ 18 MV*	*CC04 18 MV*
2×2	3×3	1	0.030	0.020	0.040	0.040
2×2	3×3	2	0.009	Low Signal	0.009	Low Signal
4×4	5×5	1	0.050	0.040	0.060	0.060
4×4	5×5	2	0.020	0.020	0.018	0.019
10×10	11×11	1	0.080	0.080	0.090	0.100
10×10	11×11	2	0.045	0.041	0.036	0.038
MLC Leakage	10×10	‐	0.016	0.013	0.017	0.013

### 5. 3D Dose Interpolation

**Figure 6:**
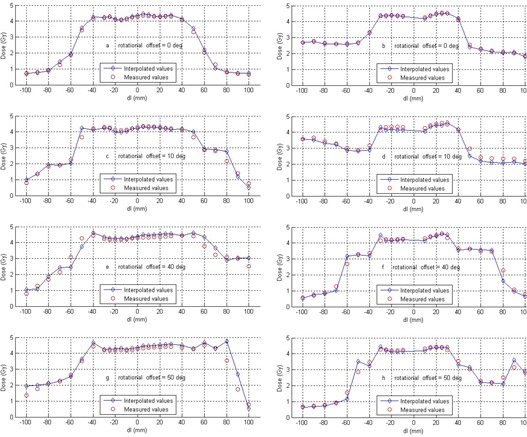
Validation of the interpolation algorithm. Figs. (a) and (b): comparison of measured and interpolated doses of an IMRT dose distribution; Figs. (c) to (h): comparison of measured and interpolated doses of the same IMRT dose distribution along the given angles through the isocenter. Each column represents one detector plane.

The interpolation algorithm was validated by visually comparing interpolated values with diode measurements (Fig. 6). A clinical IMRT plan was chosen, and the measured data were compared with the interpolated values along the central line of each detector plane (Figure 6(a) and (b)). The plan was then rotated 10°, 40°, and 50° in order to obtain pairs of measured and interpolated data along the same lines (Figs. 6(c) to 6(h)). The measured and calculated dose distributions showed excellent agreement on visual inspection. The interpolated dose profiles and the measured profiles showed good agreement, with the exception of areas where the combination of steep dose gradients and large spacing between interpolated nodes occurred.

The performance of the device was evaluated in a clinical setting by measuring the dose distribution of IMRT plans of 13 patients involving various sites. The plans were comprised of 6‐MV and 18‐MV beams and included various numbers of segments and beams, including non‐coplanar beams (Table 1). One of the central nervous system plans had beams with large couch angles; consequently, the beam passed through the top edge of the cylindrical phantom, and this plan (see the final entry in Table 1) had the lowest gamma values (57.1% for the 3%/3 mm and 77.8% for the 5%/5 mm) for the threshold analysis. This deficiency could be remedied by redesigning the phantom with a dome‐shaped top. The QA result of this plan is excluded from the following discussions. Analysis of the other 12 plans indicated that the percentage of detectors passing the ±5% dose criteria ranged from 99.9% to 58.9% with a 20% threshold, and 99.3% to 60.3% for the full analysis. The percentage of detectors with a gamma index ≤1 with the 5%/5 mm DTA criteria ranged from 100.0% to 85.5% for the 20% threshold and 99.8% to 84.8% with the full analysis. The corresponding values with 3%/3 mm criteria were 98.7% to 62.9% and 98.8% to 62.4% with and without the threshold, respectively.

**Figure 7:**
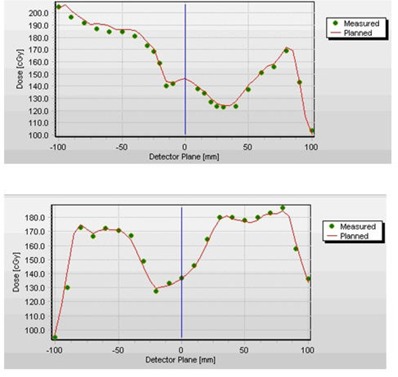
Sample of comparison of measured (points) and planned profiles (line) along the two detector planes for a clinical IMRT case.

Conventional IMRT QA results using ion chamber and film methodologies were also obtained for each plan. The direct comparison of gamma analysis with the conventional methods was not meaningful due to the difference in the size and shape of the phantoms. The conventional IMRT QA was only used to demonstrate that the plans were calculated and delivered correctly. In addition to the gamma analysis, the measured and calculated profiles were compared qualitatively (Fig. 7).

## IV. DISCUSSION

### A. Device Characterization

Our evaluation of the Delta^4^ device for IMRT QA in 3D space clearly showed that the device is capable of precise, accurate, efficient IMRT QA measurements. The high precision is possible due to the relatively high sensitivity of diodes (about 18,000 times that of an ion chamber). The high sensitivity makes it possible to design diodes with very small sensitive volume and still measure small signals from leakage and scatter radiations, which cannot be overlooked in IMRT treatments. In addition, the use of synchronization pulses limits dose measurements to the duration of the dose pulse only, thus improving the signal‐to‐noise ratio. The choice of a p‐type diode facilitates constant dose‐per‐pulse response and achieves a negligible decrease in sensitivity due to radiation damage (0.9% per KGy for 6 MV beam, <1% per kGy in 10‐MeV electron beam).^(31)^ The temperature dependence of the diodes is another potential source of uncertainty. Temperature dependence was not measured in this study; however, previous studies^(^ ^31^ ^,^ ^32^ ^)^ have shown it to be very small (i.e. 0.4%/°C). Moreover, the device's software allows for this small correction. In this study, all absolute measurements were made with the temperature dependence correction feature turned on in the software, which did not affect long‐term or short‐term reproducibility. The device compares measured absolute doses at 1069 points against treatment plan and, therefore, an independent IMRT QA can be performed with this device, in spite of its reliance on planned dose distribution in the detectorless region. In addition, it is expected that any significant delivery errors will be detected by analyzing beam‐by‐beam comparison.

### B. Clinical Characterization

The physicist has the ability to choose the orientation of the phantom, the location of the treatment isocenter with respect to the phantom, and the normalization detector for the analysis. The normalization detector is a detector chosen at or near the prescription dose level that will determine the scale for calculations of the gamma factors; it is generally the central detector. When all of these parameters were properly chosen, the clinical measurements were found to be accurate enough to use a 3%/3 mm criteria for the gamma analysis, which may be better able to identify potential problems than a 5%/5 mm criteria.^(^ ^26^ ^,^ ^33^ ^–^ ^35^ ^)^ However, it should be noted that one of the clinical plans could not be evaluated with the device at all.

As mentioned in the previous section, the phantom orientation can effectively rotate the detector planes by 10° to minimize, but not eliminate, gracing beam angles. The system's ability to correct for any remaining angular dependency was evaluated using one of the prostate plans, which was first delivered as planned with three beams gracing the detector planes at a 5° angle and then redelivered with all beams rotated by 50°. The percent of detectors with a gamma index ≤1 increased by 5% for the 5%/5 mm criteria, and by 7.6% for the 3%/3 mm criteria with the 20% threshold analysis. The corresponding increases in gamma values with the full analysis values were 2.2% and 4.5%, respectively.

The importance of choosing the appropriate normalization detector was demonstrated in a head and neck plan where isocenter was at the inferior border of the field. When the measured dose distribution was normalized at isocenter, the percentage of detectors within ±5% of the expected dose for the threshold analysis was only 67.7%. But when the data were renormalized to a detector in the center of the high‐dose region, the results improved to 98.5%. The percentage of detectors with a 3 mm/3% gamma ≤1 for the threshold analysis also increased from 87.4% to 98.5% when the normalization was done in a high‐dose region.

Delta^4^ is very flexible and will allow the treatment isocenter to be at any location with respect to the phantom. This feature provides the user with the freedom to adjust the phantom orientation to avoid beams gracing the detectors. It also offers the ability to minimize the probability that a segment would be delivered with no primary radiation reaching a detector.

A 14th plan for the treatment of mesothelioma was initially included but could not be measured. Further investigation revealed that this plan contained segments that were outside the 20 cm×20 cm detector area for several beams and, hence, the system did not measure any doses for these segments. The Delta^4^ system expects that at least one detector will receive some of the unblocked primary beam per segment or it will not continue with the measurements. The manufacturer‐recommended procedure when the field size exceeds the detector area is to offset the center of the phantom with respect to the central axis of the beam. For this plan, no shifted central axis position could be found to place at least one detector in the irradiated area for all segments of all beams.

## V. CONCLUSIONS

Full 3D IMRT QA is a laborious and time‐consuming procedure. Properly designed instrumentation and measuring devices can greatly reduce the burdens associated with this procedure. The Delta^4^ IMRT QA device has the necessary reproducibility, accuracy, stability, angular dependence, and energy response to perform 3D IMRT QA efficiently in a clinical setting. We found it to be important to renormalize the dose distribution in the high dose region and to exclude detectors that receive less than a certain minimum dose for the IMRT QA analysis. The commissioning of the device is simple and easy to implement. Automatic data acquisition and central database capability enable clinicians to perform QA efficiently. The device has built‐in tools to identify any discrepancies, so that they can be analyzed and addressed before treatment.

## ACKNOWLEDGEMENTS

The work was performed at The University of Texas M. D. Anderson Cancer Center, Houston, Texas, and was supported by National Cancer Institute Grant CA 10953. We thank the core physics team at MD Anderson Cancer Center for providing to us the conventional IMRT QA data, and Dr. Dragan Mirkovic for his assistance with programming in MATLAB. This work was presented in part at the 49th annual meeting of the American Association of Physicists in Medicine, Minneapolis, MN, in July 2007.^(36)^
